# Normalization of Spinal Cord Total Cross-Sectional and Gray Matter Areas as Quantified With Radially Sampled Averaged Magnetization Inversion Recovery Acquisitions

**DOI:** 10.3389/fneur.2021.637198

**Published:** 2021-03-25

**Authors:** Eva M. Kesenheimer, Maria Janina Wendebourg, Matthias Weigel, Claudia Weidensteiner, Tanja Haas, Laura Richter, Laura Sander, Antal Horvath, Muhamed Barakovic, Philippe Cattin, Cristina Granziera, Oliver Bieri, Regina Schlaeger

**Affiliations:** ^1^Neurologic Clinic and Policlinic, University Hospital Basel, Basel, Switzerland; ^2^Department of Clinical Research, University of Basel, Basel, Switzerland; ^3^Translational Imaging in Neurology (ThINK) Basel, Department of Medicine and Biomedical Engineering, Research Center for Clinical Neuroimmunology and Neuroscience Basel (RC2NB), University Hospital Basel and University of Basel, Basel, Switzerland; ^4^Division of Radiological Physics, Department of Radiology, University Hospital Basel, Basel, Switzerland; ^5^Department of Biomedical Engineering, University of Basel, Allschwil, Switzerland

**Keywords:** spinal cord gray matter imaging, MRI, normalization, inter-subject variability, minors, spinal muscular atrophy

## Abstract

**Background:** MR imaging of the spinal cord (SC) gray matter (GM) at the cervical and lumbar enlargements' level may be particularly informative in lower motor neuron disorders, e. g., spinal muscular atrophy, but also in other neurodegenerative or autoimmune diseases affecting the SC. Radially sampled averaged magnetization inversion recovery acquisition (rAMIRA) is a novel approach to perform SC imaging in clinical settings with favorable contrast and is well-suited for SC GM quantitation. However, before applying rAMIRA in clinical studies, it is important to understand (i) the sources of inter-subject variability of total SC cross-sectional areas (TCA) and GM area (GMA) measurements in healthy subjects and (ii) their relation to age and sex to facilitate the detection of pathology-associated changes. In this study, we aimed to develop normalization strategies for rAMIRA-derived SC metrics using skull and spine-based metrics to reduce anatomical variability.

**Methods:** Sixty-one healthy subjects (age range 11–93 years, 37.7% women) were investigated with axial two-dimensional rAMIRA imaging at 3T MRI. Cervical and thoracic levels including the level of the cervical (C4/C5) and lumbar enlargements (T_max_) were examined. SC T2-weighted sagittal images and high-resolution 3D whole-brain T1-weighted images were acquired. TCA and GMAs were quantified. Anatomical variables with associations of |*r*| > 0.30 in univariate association with SC areas, and age and sex were used to construct normalization models using backward selection with TCA_C4/C5_ as outcome. The effect of the normalization was assessed by % relative standard deviation (RSD) reductions.

**Results:** Mean inter-individual variability and the SD of the SC area metrics were considerable: TCA_C4/5_: 8.1%/9.0; TCA_Tmax_: 8.9%/6.5; GMA_C4/C5_: 8.6%/2.2; GMA_Tmax_: 12.2%/3.8. Normalization based on sex, brain WM volume, and spinal canal area resulted in RSD reductions of 23.7% for TCAs and 12.0% for GM areas at C4/C5. Normalizations based on the area of spinal canal alone resulted in RSD reductions of 10.2% for TCAs and 9.6% for GM areas at C4/C5, respectively.

**Discussion:** Anatomic inter-individual variability of SC areas is substantial. This study identified effective normalization models for inter-subject variability reduction in TCA and SC GMA in healthy subjects based on rAMIRA imaging.

## Introduction

Substantial advances in understanding spinal muscular atrophy (SMA) etiopathogenesis have catalyzed the development of novel therapeutic strategies. With the approval of the first disease-modifying treatments for SMA, the need for biomarkers that allow reliable monitoring of the disease course and the therapeutic response in SMA patients has substantially grown. Current advances in morphometric MRI development allow gray (GM) and white matter (WM) quantification in the spinal cord (SC) (1–7), which may help in improving the *in vivo* characterization of motor neuron diseases or other neurodegenerative SC diseases. Imaging the cervical and lumbar enlargements could be informative, especially in lower motor neuron diseases, e.g., SMA, or lower motor neuron-predominant amyotrophic lateral sclerosis (ALS).

Radially sampled averaged magnetization inversion recovery acquisition (rAMIRA) (8, 9) is a novel magnetic resonance imaging (MRI) approach to perform SC imaging with favorable contrast in clinical settings, which is well-suited for GM/WM quantitation not only in the cervical, but also in the thoracic SC. Briefly, after an inversion recovery preparation, rAMIRA typically acquires five radially sampled images with increasing inversion times (8). The first images of the series with shorter inversion times display high gray matter to white matter contrast, while the images with longer inversion times show a bright CSF in contrast to a dark SC (8, 9). These acquired inversion images can be combined to fine-tune and even enhance the signal-to-noise ratio (SNR) and contrast-to-noise ratio (CNR) (9). Due to the radial sampling scheme with a balanced steady-state free precession readout module, rAMIRA provides a low sensitivity to motion effects such as heartbeat and breathing, which is a crucial issue in imaging of the thoracic SC (8). Based on these advantages and a good in-plane resolution, rAMIRA images are well-suited for quantifying both GM area and total cross-sectional area (TCA) in the SC.

More recently, several semi-automated and automated tools (10–17) have been developed for segmentation of the SC GM and WM from MR images, including promising automated segmentation algorithms specifically developed for the AMIRA approach (7, 14). The reliability of the segmentation methods in single center studies is in general high, and some methods have been validated in multi-centric settings (18). Despite these substantial advances, the anatomic accuracy for delineation of SC GM is still judged based on manual algorithms (18), in particular in studies involving the thoracic SC.

Prior to applying morphometric SC techniques such as rAMIRA in larger clinical studies in lower motor neuron disorders, it is important to understand the sources of inter-subject variability of SC GM and WM area measurements in healthy subjects to increase both the sensitivity and specificity in detecting pathology-associated changes. Normalization aims to reduce biological, anatomical variation unrelated to the disease.

Previous studies in multiple sclerosis patients proposed to decrease anatomic inter-subject variability by applying normalization approaches based on correlations between the upper cervical total cross-sectional SC area/cervical SC volume and (i) skull size in healthy subjects (19, 20), (ii) lumbar enlargement SC area (21), or (iii) SC length (22, 23). However, results remain partly conflicting. Papinutto et al. (24) recently reported a 17.7% reduction of inter-subject variability in upper cervical total cross-sectional SC area at the intervertebral disc level C2/C3 based on a normalization approach combining SIENAX v-scale (25) and the product of the maximum axial anterior–posterior and lateral diameters of the cervical spinal canal in a cohort of healthy middle-aged adults based on phase-sensitive inversion recovery imaging.

The level C2/C3 has been the major target in multiple sclerosis imaging studies (26, 27) as it is clearly situated above the cervical SC enlargement and is therefore anatomically less variable than the levels below.

Nevertheless, the lower levels of the cervical and thoracic SC that contain the motor neurons to the arm and leg muscles are of special relevance to the study of lower motor neuron disorders (e.g., SMA) and have been less well-studied. There are only a few MRI studies assessing the SC of children and adolescents (28–30), none focusing on the SC GM. Children and adolescents are the leading target group for the recently approved SMA treatments; therefore, data on SC GM variations are needed to develop treatment monitoring methods.

The aims of this study were to assess the anatomic inter-subject variability in TCA and GM areas at several levels in the cervical and thoracic SC based on rAMIRA imaging in a cohort of healthy subjects with a broad age range including both adults as well as minors and to develop and evaluate potential normalization strategies for inter-subject variability reduction.

## Methods

### Participants

Sixty-one healthy subjects (range 11–93 years, mean age 46.0 years, SD 24.7, 37.7% women) including 18 minors (range 11–17 years, mean age 13.9, SD 1.9, 46.2% female) without a neurological or cognitive disease were included into the study after written informed consent was obtained. The local ethics committee approved the study.

### MRI Acquisition

**Figure 1:**
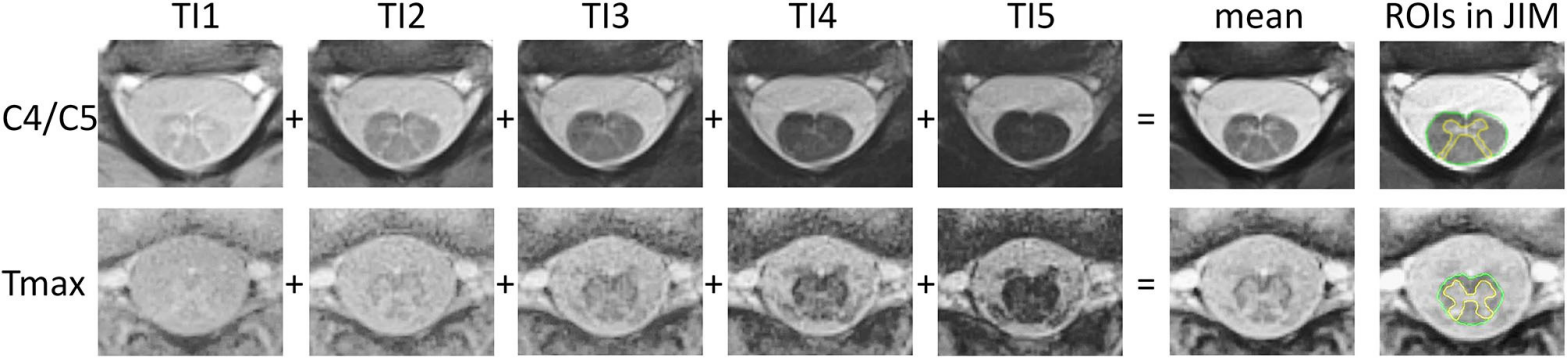
rAMIRA images of the spinal cord in an 11-year-old boy. From left to right: axial rAMIRA images at intervertebral disc level C4/C5 (top row) and T_max_ (bottom row) acquired at five inversion times (TI), mean image (combination of the images at five TIs); and mean images with the region of interests (ROIs) spinal cord total cross-sectional area (TCA, green) and gray matter area (GMA, yellow) which were segmented using the software JIM.

All participants were examined with the same 3T whole-body MR system (Siemens Magnetom PRISMA, Siemens Healthineers, Erlangen, Germany) using a 64-channel head and neck coil and the built-in spine coil for reception. Axial two-dimensional rAMIRA images (8, 9) were acquired perpendicular to the SC at the intervertebral disc levels C2/C3, C3/C4, C4/C5, C5/6, T9/T10, and T_max_ [level of the lumbar enlargement, which was identified by visual inspection on the corresponding sagittal and coronal T2-weighted turbo spin echo images of the spine (cf. below) by TH (>20 y of experience)]. The employed rAMIRA acquisition protocol was identical to the “optimized standard protocol version” presented in the corresponding methods paper (8). Thus, the relevant sequence parameters for rAMIRA were: field of view = 128 × 128 mm^2^, 512 readout samples (includes 2 × oversampling), 260 projections, isotropic in-plane resolution 0.50 × 0.50 mm^2^, slice thickness 8 mm, bandwidth = 310 Hz/Px, flip angle = 50 deg, signal averaging = 2. Five images with the mean inversion times TI_eff_ = 174, 239, 304, 368, 433 ms were acquired simultaneously and later combined (cf. below and Figure 1). The sequence uses cardiac triggering to mitigate potential pulsation artifacts, which was realized with a standard infrared finger clip (simple pulse triggering). Hence, for a heart rate of 60 bpm, rAMIRA's acquisition time corresponds to 2:39 min per slice, for instance.

Furthermore, all participants received T2-weighted turbo spin echo imaging covering the whole SC in sagittal and coronal slice orientation. Here, the most relevant sequence parameters were [1] sagittal: in-plane resolution = 0.7 × 0.7 mm^2^, 17 slices of thickness 3 mm, TR = 3,400 ms, TE = 102 ms; [2] coronal: in-plane resolution = 1.4 × 1.4 mm^2^, 17 slices of thickness 3 mm, TR = 3,500 ms, TE = 95 ms. Additionally, 3D isotropic high-resolution whole-brain T1-weighted images were acquired with the magnetization prepared rapid gradient echo (MPRAGE) sequence, using the following parameters: 1.0 mm isotropic resolution, TI = 1,100 ms, TR = 2,000 ms, TE = 2.12 ms, flip angle = 8 deg, matrix = 256 × 256 × 192.

### MRI Analysis

All rAMIRA images were visually inspected for image quality [by TH (>20 y of experience), EK (1 y), MJW (3 y), and CW (>20 y)]. Only images with sufficient quality were segmented. Segmentations were not possible in 3 out of 244 images in the cervical SC and in 12 out of 122 in the thoracic SC (for details s. results section). For the quantitation of the GM area and TCA, one mean image of all five simultaneously acquired inversion images of the rAMIRA series was calculated, which shows a high gray matter to white matter contrast, as well as sufficient contrast between SC and CSF (Figure 1) (8, 9).

Total cross-sectional areas were segmented in a semi-automated way using the software JIM 7 (http://www.xinapse.com) (31). Following a previously published reliable segmentation algorithm (5, 6, 26), GM was segmented manually three times by one single rater and the mean was calculated (by MJW).

Brain T1-weighted images were investigated using SPM12 (https://www.fil.ion.ucl.ac.uk/spm/) to determine the total intracranial volume (TIV; a frequently used normalization parameter for brain volumes) (32) as well as brain GM and WM volumes.

**Figure 2:**
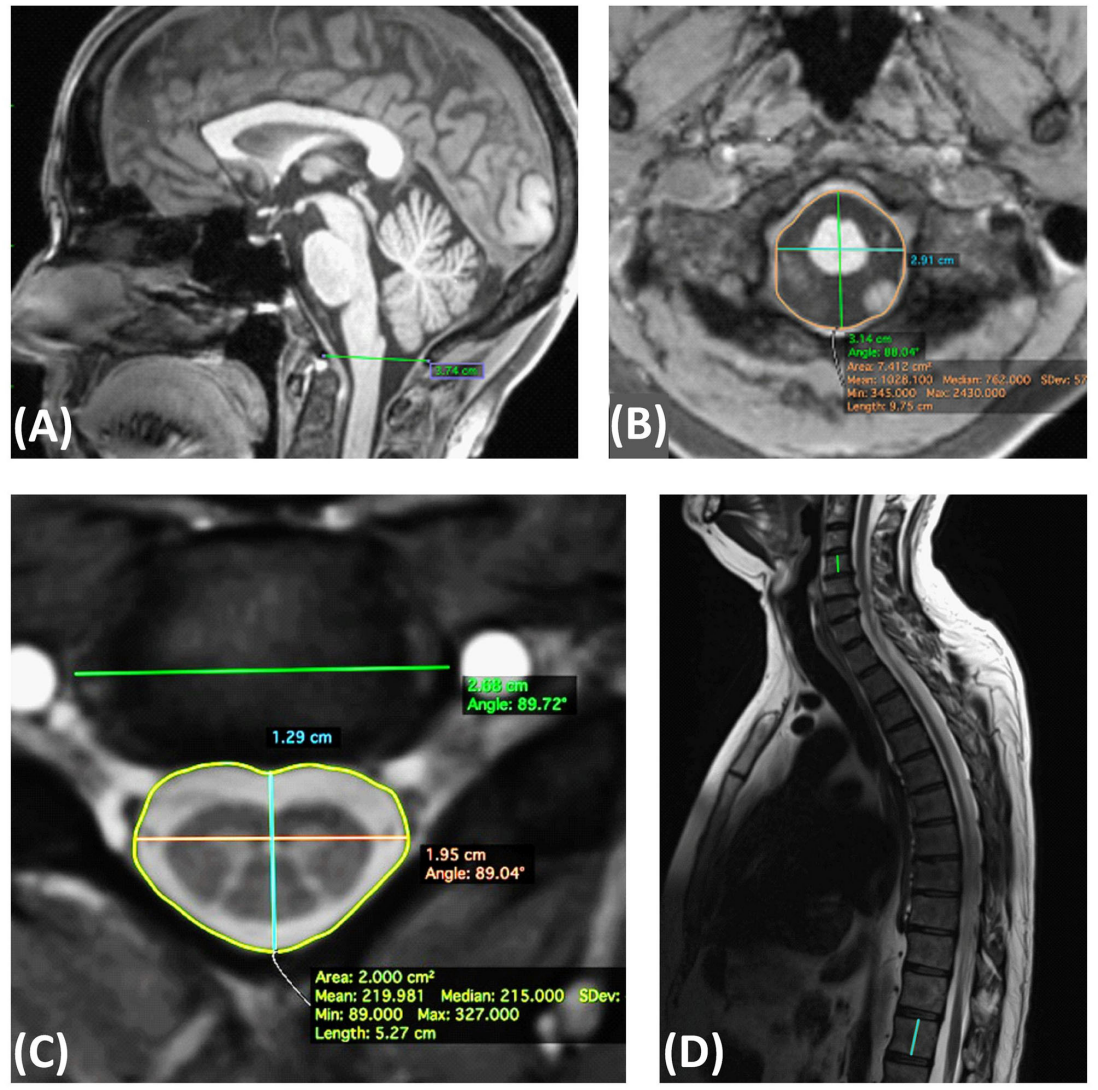
Definition of the skeletal metrics. **(A)** Sagittal T1 weighted MPRAGE: McRae line (distance between Basion–Opisthion). **(B)** Axial MPRAGE: anterior posterior and lateral diameters, as well as area of the foramen magnum. **(C)** Axial rAMIRA: Maximum axial anterior posterior and lateral spinal canal diameters, spinal canal area, maximum lateral vertebral body width. **(D)** Sagittal T2 weighted turbo spin echo: middle vertebra height C4 and T12.

In addition, the following parameters were determined as potential normalization factors at the levels C4/C5 and Tmax (Figure 2) (by EK):

- Maximum axial anterior–posterior and lateral spinal canal diameters, axial spinal canal area, maximum axial lateral vertebral body width on the axial two-dimensional rAMIRA images; additionally, the product of the anterior–posterior and lateral spinal canal diameter was calculated at both levels.
- Middle vertebra height C4 and T12 on the T2-weighted sagittal images.
- Anterior–posterior and lateral diameters, as well as area of the foramen magnum, and distance between Basion–Opisthion on an isotropic, high-resolution 3D whole-brain T1-weighted image. The product of the anterior–posterior and lateral diameter of the foramen magnum was calculated as well.

The inter-rater reliability of the manually assessed anatomical parameters was determined in 14 datasets by two independent operators (by EK and CW) and showed excellent reliability with intra-class correlation coefficients (ICCs, two-way random, absolute agreement) (33) >0.96, as listed in the supplement (Supplementary Table 1). Segmentation was done with OsirixLite (https://www.osirix-viewer.com/).

### Statistical Analysis

Statistical analysis was performed using IBM SPSS Statistics for Macintosh, Version 25.0, Armonk, NY: IBM Corp., and JMP pro, Version 14. SAS Institute Inc., Cary, NC, 1989-2019.

- Inter-Individual Variability Assessments

To assess the inter-individual variability of SC areas, the respective deviation from the group mean was calculated for each subject as

$$\begin{array}{l} \frac{\left|\text{measured area }-\text{ mean area}\right|}{\text{mean area}}*100\left[\text{\%}\right] \end{array}$$

- Assessing the Effect of Age and Sex

Differences in rAMIRA-based SC areas between men and women were assessed using linear regression analysis co-varying for age.

Linear and quadratic fits for (a) TCA and (b) SC GM areas vs. age were assessed for the whole study group (*n* = 61), and the *r*^2^ of the models was reported. For practicability reasons to facilitate future analyses in specific target populations, we also assessed SC areas in three nearly equally sized sub-groups: minors (Group 1: aged <18 years, *n* = 18), middle-aged (Group 2: aged 18–65 years, *n* = 23) and elderly subjects (Group 3: aged >65 years, *n* = 20) using linear regression analysis with sex as additional covariate, respectively.

- Normalization Models

For the development of the normalization models, the associations of the anatomical parameters with rAMIRA-based SC areas at C4/C5 and T_max_ were first assessed using Pearson correlation coefficients since all variables were normally distributed. Bonferroni correction was performed with a correction factor of 17 (*p* < 0.05/17) to correct for *n* = 17 tests. This analysis was performed using the data from all subjects (*n* = 61).

We then performed a backward selection procedure starting with a model containing all anatomical variables with a Pearson correlation coefficient of |*r*| > 0.30 (34) in univariate analysis as well as age and sex as predictor variables and TCA at the level C4/C5 as outcome parameter.

This procedure was performed (a) considering brain GM and WM volumes as potential predictors (approach suitable for studies in healthy controls and diseases known to not affect brain GM and/or WM volumes) and (b) without considering brain GM and WM volumes (approach suitable for studies in diseases known to affect these brain volumes).

The adjusted *r*^2^ of the models resulting from the backward selection was reported.

For normalization we used the approach described by Sanfilipo and Papinutto (25, 35):

$$\begin{array}{l} {\text{Area}}_{\text{predicted}}={\text{Area}}_{\text{measured}}+\text{a}\left({\text{X}}_{\text{mean}}-{\text{X}}_{\text{measured}}\right)\\ +\text{ b}\left({\text{Y}}_{\text{mean}}-{\text{Y}}_{\text{measured}}\right)+\text{c}\left({\text{Z}}_{\text{mean}}-{\text{Z}}_{\text{measured}}\right) \end{array}$$

with a, b, and c being the estimates (regression coefficients) obtained by the linear regression analysis for the predictor variables surviving the backward selection procedure and X, Y, Z the measured values of these variables.

The performance of the resulting normalization models was expressed a) as the % reduction of inter-individual variability of the normalized SC areas of each model in relation to the variability of the non-normalized areas and b) as the % relative standard deviation (% RSD) reductions of the predicted areas to the % RSD of the non-normalized, measured areas. The relative standard deviation (RSD) is the standard deviation divided by the mean SC area.

The normalization model with the largest relative inter-individual variability reduction was then applied to all other SC level measurements.

In analogy, we developed a normalization model for the sub-group of minors (*n* = 18).

## Results

The acquired rAMIRA images displayed a good quality in general. Segmentations were not possible in 1.2% of the images in the cervical SC (due to motion artifacts in minors) and in 9.8% in the thoracic SC due to image artifacts originating from flow in near-by large vessels or due to artifacts originating from cardiac and breathing motion.

### Inter-individual Variability of Spinal Cord Area Measurements in Healthy Subjects

The inter-individual mean relative variability for TCA and GM area, as well as the % relative SD (% RSD: defined as the SD divided by the group mean), are summarized in Table 1.

**Table 1:** Mean % inter-individual variability, mean, SD and % RSD (% relative SD) of spinal cord total cross-sectional areas (TCA) and gray matter areas (GMA) at the intervertebral disc levels C2/C3–C5/C6, T9/T10, and T_max_ (level of the lumbar enlargement) of the whole study population.

**Level**		**Mean (in mm^**2**^)**	**SD**	**% RSD**	**Mean % inter-individual variability**
C2/C3	TCA	83.2	7.5	9.0	6.6
	GMA	15.5	1.4	8.9	7.0
C3/C4	TCA	87.4	8.31	9.5	7.1
	GMA	19.1	2.0	10.4	8.2
C4/C5	TCA	88.5	9.0	10.1	8.1
	GMA	20.2	2.2	10.7	8.6
C5/C6	TCA	85.2	8.0	9.3	7.4
	GMA	20.0	2.2	11.1	9.0
T9/T10	TCA	46.8	4.4	9.3	7.3
	GMA	9.80	1.1	11.4	8.9
T_max_	TCA	62.9	6.5	10.4	8.9
	GMA	24.0	3.8	15.6	12.2

### Effects of Age and Sex

Effects of age and sex are summarized in the supplement in Supplementary Tables 2, 3.

In brief, men showed significantly larger SC GM areas at C3/C4 (*p* = 0.0350) and at T_max_ (*p* = 0.0497) than women. At all other levels, we detected no significant area differences between sexes (Supplementary Table 2).

The linear and quadratic fits for TCA and GM areas vs. age for the whole group showed in general relatively low *r*^2^ indicating low accuracy of the models: *r*^2^ (linear/quadratic fit) TCA_C2/C3_: 0.009/0.088; TCA_C3/C4_: 0.005/0.049; TCA_C4/C5_: 0.001/0.031; TCA_C5/C6_: 0.010/0.034; TCA_T9/T10_: 0.041/0.101; TCA_Tmax_: 0.000/0.014; GM area_C2/C3_: 0.051/0.055; GM area_C3/C4_: 0.000/0.017; GM area_C4/C5_: 0.009/0.018; GM area_C5/C6_: 0.038/0.045; and GM area_T9/T10_: 0.229/0.24, GM area_Tmax_: 0.13/0.137.

For reasons of practicability, we also analyzed SC areas in age sub-groups of minors, middle-aged, and elderly subjects with adjustment for sex. These results are summarized in Supplementary Table 3. Maximum mean TCA values were consistently observed in the sub-group of middle-aged subjects at all levels, with TCA differences being significant between minors and middle-aged subjects at the level C2/C3 (mean difference 4.78 mm^2^, SE 2.34, 95% CI of the difference: 0.09–9.47, *p* = 0.0457), and between middle-aged and elderly subjects at the level T9/T10 (mean difference 3.30 mm^2^, SE 1.28, 95% CI of the difference: 0.72–5.88, *p* = 0.0131).

While minimum mean GM area values were consistently observed in the subgroup of elderly subjects at all levels, maximum mean GM area values were observed in the subgroup of middle-aged subjects only at the level C3/C4 (Supplementary Table 3). Significant GM area differences could be detected between middle-aged and elderly subjects at the level C2/C3 (mean difference 0.83 mm^2^, SE 0.41, 95% CI of the difference: 0.00–1.66, *p* = 0.0495) and between minors and middle-aged subjects at the level T9/T10 (mean difference 0.80 mm^2^, SE 0.37, 95% CI: 0.06–1.55, *p* = 0.0353) (Supplementary Table 3).

### Normalization Models

Among the chosen metrics, TIV, brain WM volume, anterior–posterior spinal canal diameter, and area of the spinal canal, as well as the product of the anterior–posterior and lateral spinal canal showed a correlation with TCA at the level C4/C5 with Pearson correlation coefficients of |*r*| > 0.30 (Table 2), with the other parameters showing lower correlation coefficients. After Bonferroni correction, none of the investigated metrics did show a significant association with SC areas at T_max_ (Supplementary Table 4); thus, normalization model development was primarily focused on the cervical SC.

**Table 2:** Associations between the anatomical metrics and total cross-sectional area (TCA) and gray matter area (GMA) at the C4/C5 level using Pearson correlation coefficients.

**Whole study population (*n* = 61)**	**TCA C4/C5**		**GMA C4/C5**	
**Metric**	***p-*Value**	**Pearson correlation coefficient**	***p*-Value**	**Pearson correlation coefficient**
CAN_C4/C5_ap	**0.000**	**0.43**	**0.000**	**0.47**
CAN_C4/C5_lat	0.470	0.26	0.178	0.18
CAN_C4/C5_area	**0.000**	**0.44**	**0.001**	**0.43**
Prod_CAN_C4/C5_ap*lat	**0.000**	**0.45**	**0.000**	**0.44**
VBW_C4/C5_lat	0.706	0.05	0.315	−0.13
VBH_C4	0.519	0.08	0.860	0.02
McRae	0.532	0.08	0.760	0.04
ForMag_ap	0.943	0.01	0.821	0.03
ForMag_lat	0.998	0.00	0.493	0.09
ForMag_area	0.377	0.12	0.201	0.17
Prod_ForMag_ap*lat	0.992	−0.00	0.892	−0.02
TIV	0.012	**0.32**	0.074	0.23
Brain GM volume	0.394	0.11	0.175	0.18
Brain WM volume	**0.000**	**0.48**	0.048	0.26
Height	0.197	0.17	0.650	0.06
Weight	0.170	0.19	0.211	0.16
BMI	0.296	0.14	0.155	0.18

P-values surviving the Bonferroni correction are bolded (p < 0.0029). CAN_C4/C5_ap, anterior–posterior diameter of the spinal canal at the level C4/C5. CAN_C4/C5_lat, lateral diameter of the spinal canal at the level C4/C5. CAN_C4/C5_area, area of the spinal canal at the level C4/C5; Prod_CAN_C4/C5_ap*lat, product of the anterior-posterior and lateral diameter of the spinal canal at the level C4/C5; VBW_C4/C5_lat, maximum vertebra body width at the level C4/C5; VBH_C4, middle vertebral body height of C4; McRae, McRae line (distance between Basion–Opisthion). ForMag_ap, anterior posterior diameter of the foramen magnum; ForMag_lat, lateral diameter of the foramen magnum; ForMag_area, area of the foramen magnum; Prod_ForMag_ap*lat, product of the anterior–posterior and lateral diameter of the foramen magnum; TIV, total intracranial volume; GM, gray matter; WM, white matter; BMI, body mass index.

The backward selection procedure yielded a model with sex, brain WM volume, and the area of the spinal canal at the level C4/C5 as predictor variables for TCA at C4/C5 as outcome (Model 1) (Table 3). Age was not a predictor variable.

**Table 3:** Linear regression analysis with total cross-sectional area (TCA) at the C4/C5 level as outcome and normalization variables after backward selection; corresponding models with gray matter area (GMA) as outcome.

**Whole study population**	**TCA C4/C5**			**GMA C4/C5**		
	***p***	**Adjusted *r*^**2**^**	**Estimate**	***p***	**Adjusted *r*^**2**^**	**Estimate**
Model 1:	<0.0001	0.43		0.0014	0.20	
Sex	0.0082		−2.8534	0.4019		−0.2523
WM volume	<0.0001		0.0861	0.0495		0.0096
CAN_C4C5_area	<0.0001		0.1235	0.0008		0.0266
Model 1a:	0.0005	0.23		0.0057	0.15	
Sex	0.0975		−2.1357	0.5926		−0.1727
TIV	0.0271		0.0177	s 0.3237		0.0020
CAN_C4C5_area	0.0010		0.1063	0.0024		0.0247
Model 2:	0.0004	0.18		0.0006	0.17	
CAN_C4C5_area	0.0004		0.1094	0.0006		0.0257

*CAN_C4C5_area, spinal canal area at the level C4/C5; TIV, total intracranial volume*.

Normalization strategies that are based on brain WM or GM volumes can be used in healthy subjects, but are not well-suited in diseases with potential changes in WM or GM volumes caused by the underlying disease itself (e.g., gray matter pathology in ALS, SMA; or mainly white matter pathology in adrenomyeloneuropathy).

We, therefore, performed a second backward selection—without considering brain GM and WM volumes—yielding a model (in analogy to Model 1) with sex, TIV, and the area of the spinal canal at the level C4/C5 as predictors for TCA at C4/C5 as outcome, permitting variables with *p* < 0.1 (Model 1a). This model was further simplified using backward selection to a model only containing the area of the spinal canal at the level C4/C5 as univariate predictor (Model 2) (Table 3).

The effect of the normalization of TCA and GM areas at the level C4/C5 based on Models 1, 1a, and 2 on the mean inter-individual variability and % relative standard deviation is summarized in Table 4.

**Table 4:** % relative standard deviation (RSD, standard deviation divided by the mean area), relative % RSD reduction, mean % inter-individual variability [(measured area in a given subject–group mean area)/group mean area], and relative % inter-individual variability reduction with respect to the measured total cross-sectional cord area (TCA) and gray matter area (GMA) at the intervertebral disc level C4/C5 for normalizations based on Models 1, 1a, and Model 2.

**Area**		**Non-normalized**	**Model 1**	**Model 1a**	**Model 2**
TCA C4/C5	% RSD	10.1	7.7	8.8	9.1
	Relative % RSD reduction [%]	–	(23.7)	(12.8)	(10.2)
	Mean % inter-individual variability	8.1	5.9	7.0	7.3
	Relative %variability reduction [%]		(27.2)	(12.9)	(10.0)
GMA C4/C5	% RSD	10.7	9.4	9.6	9.7
	Relative % RSD reduction [%]	–	(12.0)	(10.0)	(9.6)
	Mean % inter-individual variability	8.6	7.5	7.9	7.9
	Relative %variability reduction [%]	–	(13.2)	(8.1)	(8.6)

*Whole study population, n = 61*.

**Figure 3:**
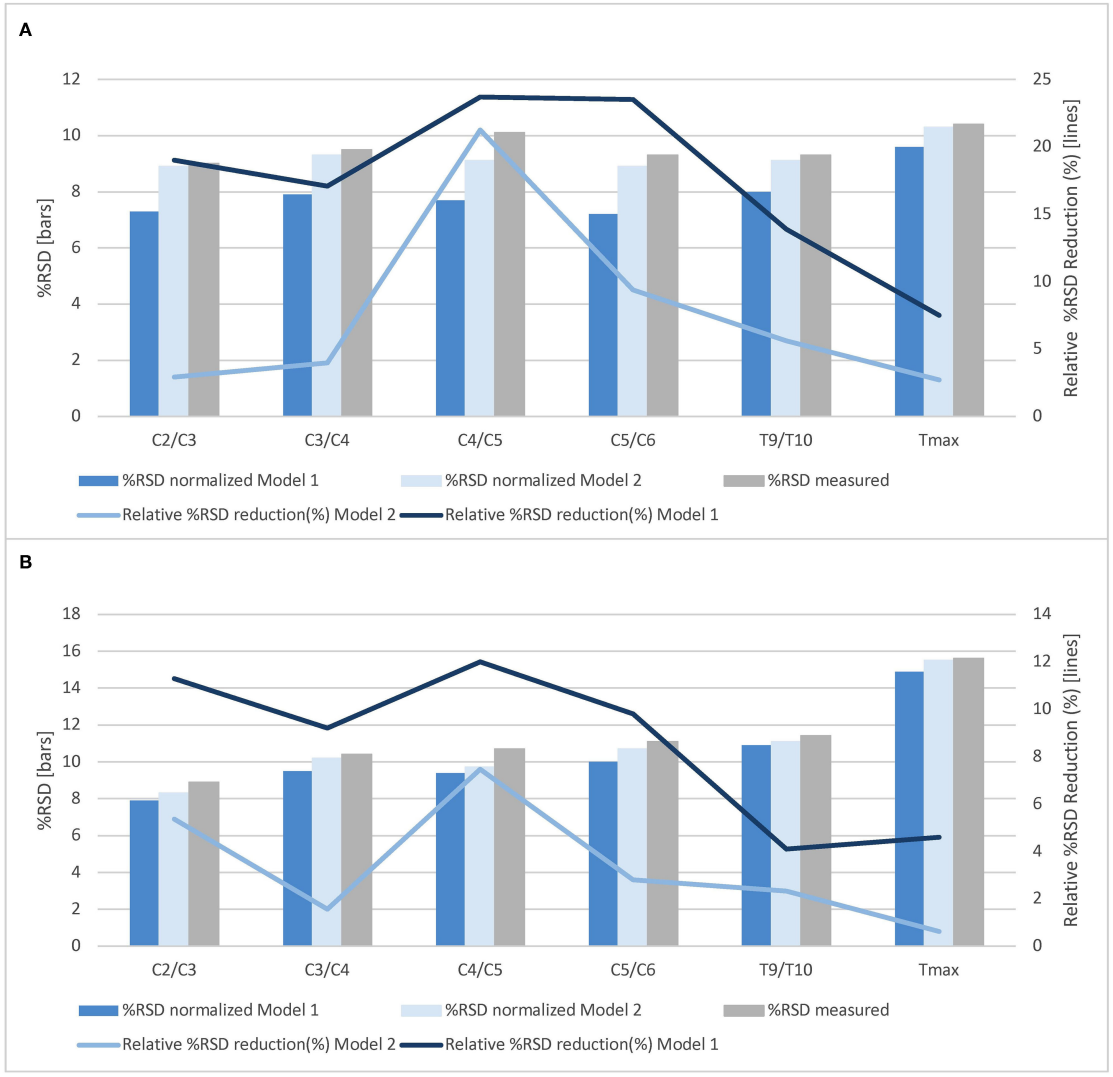
Normalization effects of Model 1 and Model 2 on total cross-sectional cord areas and gray matter areas at all cord levels. The % relative standard deviation (RSD, standard deviation divided by the mean area) and relative % RSD reduction obtained by normalization based on Model 1 (sex, brain white matter volume and spinal canal area at the level C4/C5) as well as by Model 2 (spinal canal area at the level C4/C5) for **(A)** total cross-sectional cord areas and **(B)** spinal cord gray matter areas (whole study population, *n* = 61).

Model 1 and Model 2 were then applied to all SC level measurements (Table 5, Figure 3).

**Table 5:** Performance of normalization by Model 1 and Model 2 for total cross-sectional cord areas (TCA) and gray matter areas (GMA) at all other cord levels.

**Level**		**% RSD measured**	**Model 1**	**Model 2**
			**% RSD normalized [relative % RSD reduction (%)]**	**% RSD normalized [relative % RSD reduction (%)]**
C2/C3	TCA	9.0	7.3 (19.0)	8.9 (1.4)
	GMA	8.9	7.9 (11.3)	8.3 (6.9)
C3/C4	TCA	9.5	7.9 (17.1)	9.3 (1.9)
	GMA	10.4	9.5 (9.2)	10.2 (2.0)
C4/C5	TCA	10.1	7.7 (23.7)	9.1 (10.2)
	GMA	10.7	9.4 (12.0)	9.7 (9.6)
C5/C6	TCA	9.3	7.2 (23.5)	8.9 (4.5)
	GMA	11.1	10.0 (9.8)	10.7 (3.6)
T9/T10	TCA	9.3	8.0 (13.9)	9.1 (2.7)
	GMA	11.4	10.9 (4.1)	11.1 (3.0)
T_max_	TCA	10.4	9.6 (7.5)	10.3 (1.3)
	GMA	15.6	14.9 (4.6)	15.5 (0.8)

*% relative standard deviation (RSD) and relative % RSD reduction obtained by normalization based on Model 1 (sex, brain WM volume and spinal canal area at the level C4/C5) as well as by Model 2 (spinal canal area at the level C4/C5) (whole study population, n = 61)*.

### Normalization Models for Minors

In a subgroup analysis, we separately examined potential normalization variables for anatomical variability reduction in minors (*n* = 18) at the C4/C5 level. Supplementary Table 5 in the supplement shows the univariate associations between SC areas and potential covariates using Pearson correlation.

Backward selection (starting with a model containing all variables with a Pearson correlation coefficient of |*r*| > 0.30 in univariate analysis together with age and sex as predictor variables and TCA at C4/C5 as outcome parameter) resulted in a model with the area of the spinal canal at level C4/C5 and weight as predictor variables (Model 3 m) (*p* of the model = 0.0027, adjusted *r*^2^ = 0.48). Normalization reduced the RSD about 32.5% for TCA and 18.3% for GM area at the level C4/C5. Inter-individual variability was reduced from 8.1 to 5.7% for TCA and reduced from 9.3 to 7.1% for GM area. This normalization model was applied to all other measured SC levels (Table 6).

**Table 6:** Performance of normalization by Model 3 m and Model 2 m for total cross-sectional cord areas (TCA) and gray matter areas (GMA) at all other cord levels in minors.

**Minors level**		**% RSD measured**	**Model 3 m**	**Model 2 m**
			**% RSD normalized [relative % RSD reduction (%)]**	**% RSD normalized [relative % RSD reduction (%)]**
C2/C3	TCA	9.1	7.0 (23.1)	7.5 (17.6)
	GMA	10.1	6.8 (32.8)	6.9 (31.9)
C3/C4	TCA	9.8	6.7 (31.8)	7.9 (18.9)
	GMA	10.9	8.7 (20.7)	9.3 (15.4)
C4/C5	TCA	11.3	7.6 (32.5)	9.0 (20.6)
	GMA	11.4	9.3 (18.3)	9.7 (14.5)
C5/C6	TCA	9.3	5.0 (45.9)	6.1 (34.5)
	GMA	11.6	7.6 (34.4)	8.5 (26.3)
T9/T10	TCA	9.0	6.2 (31.9)	8.0 (12.0)
	GMA	6.5	5.1 (20.3)	5.3 (17.2)
T_max_	TCA	9.6	8.8 (9.2)	9.6 (0.4)
	GMA	10.1	8.2 (19.3)	8.5 (16.3)

*% relative standard deviation (RSD) and relative % RSD reduction of normalization by Model 3 m (spinal canal area at the C4/C5 level and weight) and Model 2 m (spinal canal area at the C4/C5 level) applied to all other measured cord levels in minors*.

For reasons of practicability, Model 3 m was further simplified to a univariate model containing the area of the spinal canal of the C4/C5 level as single predictor variable in minors (in analogy to Model 2 named Model 2 m) (*p* = 0.0074, adjusted *r*^2^ = 0.33). This normalization model was also applied to all other measured SC levels in minors (Table 6).

## Discussion

Anatomical inter-subject variations in healthy subjects are a relevant source of SC TCA and GM area variability. Our study demonstrated an inter-individual variability of TCA and SC GM areas ranging from 6 to 9% in the cervical and 9–12% in the thoracic SC, which is in line with prior reports investigating the upper cervical SC (24).

Before applying morphometric SC GM/WM imaging techniques such as the novel rAMIRA approach for atrophy assessments in clinical studies in lower motor neuron disorders, it therefore seems necessary to develop efficient strategies to reduce the inter-subject variability of SC area measurements in healthy subjects to facilitate the detection of pathology-related changes.

This study explored potential normalization strategies for variability reduction in 61 healthy subjects with a broad age range from 11 to 93 years (including a subgroup of 18 minors).

Normalization models were developed using backward selection starting with a model containing the predictor variables age and sex as well as all anatomical metrics with a Pearson correlation coefficient of |*r*| > 0.3 in univariate analysis with TCA at C4/C5—the level of the cervical enlargement—as outcome. The backward selection procedure yielded a model including the predictor variables sex, spinal canal area at C4/C5, and brain WM volume. Normalization of SC areas by this model reduced the % RSD of TCA at the level C4/C5 by 24%, of GM area by 12%, and also at the other cervical SC levels in the range of 17–24% (TCA) and 9–12% (GM area). Therefore, this approach seems an effective normalization strategy suited for studies in healthy subjects or in SC diseases that do not affect cerebral WM.

However, as brain WM volume is frequently altered in SC diseases either directly by a disease pathology affecting both brain and SC (e.g., in multiple sclerosis) or potentially also secondary to lower motor neuron disorders, e.g., by retrograde trans-synaptic degeneration, we also performed a second backward analysis containing only demographic and skeleton-based variables otherwise following the above mentioned selection criteria. The resulting model contained the spinal canal area as single predictor. Normalization by this model reduced the % RSD of TCA and GM area at C4/C5 by 10%, respectively. However, application of this normalization to other SC levels, particularly to the thoracic SC, showed only small effects in our cohort, with relative % RSD reductions ranging from 1 to 7%.

Age was not a significant predictor variable for SC areas in this study. During normalization model development, age consistently was eliminated in the backward selection process. We consistently observed maximum mean TCA values in the middle-aged group at all SC levels. This is in line with the observation by Papinutto et al. (24) of a TCA peak at the level C2/C3 at ~45 years of age. While at all levels minimum mean SC GM area values were observed in the subgroup of elderly subjects, maximum mean SC GM area values were observed in the subgroup of middle-aged subjects only at the level C3/C4, with equally high or higher mean SC GM area values in minors at all other levels.

Since children and adolescents are the main target group for the recently approved SMA treatments (36) and no data exists on SC GM area inter-subject variability, we conducted a sub-group analysis in minors (*n* = 18). Following the same variable selection procedure as described above, normalization based on the spinal canal area at the level C4/C5 and the variable body weight resulted in a reduction of % RSD of TCA values at the level C4/C5 by 32%. Analogous normalization of GM areas reduced the % RSD by 18% at the level C4/C5, and also consistently at all other cervical and thoracic levels by 19–34%. The % RSD reduction observed in TCAs by this normalization method are slightly larger than what has been described in a very recently published study in minors aged 7–17 years by normalization with the product of the anterior posterior and lateral diameter of the spinal canal at the level C2/3 and skull volume (30). This difference could be partly explained by difference in in-plane resolution of the images used for SC segmentation (0.5 vs. 1 mm^2^) (30) and by the chosen predictors. Effects on SC GM area were not investigated in that study (30).

The spinal canal area is relatively easy to measure and showed excellent inter-rater reliability coefficients (33). Normalization based on spinal canal area at the level C4/C5 and body weight could therefore be a promising and brain volume-independent procedure in upcoming studies involving minors with lower motor neuron diseases to reduce anatomical inter-subject variability. Whether this approach increases the sensitivity and specificity in detecting disease-related changes in motor neuron disorders or treatment effects, needs to be further investigated in subsequent studies involving patients.

Image quality of the cervical rAMIRA images was generally high permitting SC, TCA, and GM segmentation in >98% of acquired cervical SC images of the study population. Imaging the thoracic SC is in general more challenging: Factors, that can negatively impact image quality at the thoracic levels include lower coil sensitivity in this area (Figure 1, bottom row) and potential artifacts arising from heart and breathing motion, from pulsating large blood vessels, and from increased susceptibility variations. Despite these limitations, the novel rAMIRA method enabled TCA and GM area segmentation in >90% of the acquired thoracic SC images. Segmentation was not reliably possible at the level T9/T10 and T_max_ in 6 out of 61 subjects, respectively, because of the upper mentioned issues. The % RSD of the SC metrics was higher at T_max_ compared to the cervical levels (Table 1), which is likely due to the high inherent anatomical variability in this region. None of the investigated regional anatomical metrics did show a significant association with SC areas at T_max._ A unifying normalization concept based on normalization of SC areas at different levels, by one model based on anatomical metrics measured at one selected level, and not by several models with regional normalization metrics, was therefore chosen. Though not perfect, this approach will also permit comparison of atrophy effects between levels.

It should be mentioned that the study has the following limitations: the sample size of 61 healthy subjects, including only 18 minors, is rather small. The assumption that the investigated skeleton-derived metrics are fully disease independent might be questioned, as secondary orthopedic complications, immobilization, and also treatment effects might alter spine metrics in a way that is not easily predicted. Spine-derived metrics were assessed manually in this study but showed excellent inter-rater reliability.

In conclusion, rAMIRA imaging is a novel approach to perform SC imaging with favorable contrast in clinical settings that is well-suited for GM/WM quantitation in both the cervical and thoracic SC. Our study demonstrates that the inter-individual variability of SC area measurements in healthy subjects is relatively high but can be effectively reduced in the cervical and to a lesser degree also in the thoracic SC, particularly in minors, by a model based on spinal canal area at the level C4/C5 and weight. By reducing anatomical inter-subject variability, these approaches may facilitate the detection of pathology-related changes and the improvement of therapeutic monitoring in lower motor neuron disorders in the future.

## Data Availability Statement

Upon reasonable request, we will render the detailed results derived from the reported analyses available.

## Ethics Statement

The studies involving human participants were reviewed and approved by Ethikkommission Nordwest- und Zentralschweiz - EKNZ. Written informed consent to participate in this study was provided by the participants or the participants' legal guardian/next of kin.

## Author Contributions

EK, MW, CW, OB, and RS conceptualized and designed the study. MW, CW, AH, PC, and OB handled the methods development. EK, JW, LR, TH, and RS collected the data. EK, JW, CW, TH, MB, LS, and RS conducted the data analysis. EK, MW, CW, and RS were in charge of the paper drafting. EK, JW, MW, CW, TH, LR, LS, AH, MB, PC, CG, OB, and RS reviewed the manuscript. OB and RS obtained the funding. All authors contributed to the article and approved the submitted version.

## Conflict of Interest

The authors declare that the research was conducted in the absence of any commercial or financial relationships that could be construed as a potential conflict of interest.
