# Supplementary material for: Normalization of Spinal Cord Total Cross-Sectional and Gray Matter Areas as Quantified With Radially Sampled Averaged Magnetization Inversion Recovery Acquisitions
Source: Front Neurol. 2021 Mar 25;12:637198. doi: 10.3389/fneur.2021.637198 (PMC8027254; doi:10.3389/fneur.2021.637198)
Supplement: Supplementary file 1 [file Data_Sheet_1.PDF]

Supplementary Material:

Supplementary Table 1: Intra-class correlation coefficients for assessment of inter-rater reliability (two- way random, absolute agreement, average measurements) of the metrics that were used for further normalization.

	CAN_C4/C5_ap	CAN_C4/C5_lat	CAN_C4/C5_area	Prod_CAN_C4/C5
**ICC**	0.996	0.968	0.994	0.993
**95%-CI**	0.984-0.999	0.904-.99	0.957-0.998	0.977-0.998

CAN_C4/C5 ap: anterior-posterior diameter of the spinal canal at the level C4/C5. CAN_C4/C5_lat: lateral diameter of the spinal canal at the level C4/C5. CAN_C4/C5_area: area of the spinal canal at the level C4/C5. Prod_CAN_C4/C5: product of the anterior-posterior and lateral diameter of the spinal canal at the level C4/C5. ICC: intra-class correlation coefficient. CI: Confidence interval.

Supplementary Table 2: Total cross-sectional area (TCA) and gray matter areas (GMA) (in mm^2) at the intervertebral disc levels C2/C3, C3/C4, C4/C5, C5/C6, T9/T10, and T_{max} (level of the lumbar enlargement) in men and women. Means are least square means with adjustment for age.

**Whole study population**		Men		Women		t-Test				
		**Mean area (mm^2)**	**SE**	**Mean area (mm^2)**	**SE**	**Differ-ence**	**SE**	**p-value**	**Lower 95% CI**	**Upper 95% CI**
**C2/C3**	TCA	83.71	1.23	82.36	1.62	1.35	2.03	0.5083	-2.72	5.43
	GMA	15.63	0.22	15.27	0.29	0.36	0.37	0.3304	-0.37	1.09
**C3/C4**	TCA	87.89	1.37	86.59	1.75	1.3	2.22	0.561	-3.15	5.75
	GMA	19.50	0.32	18.39	0.41	1.11	0.51	**0.0350**	0.08	2.14
**C4/C5**	TCA	89.09	1,47	87.48	1.89	1.61	2.39	0.5048	-3.19	6.40
	GMA	20.47	0.35	19.82	0.45	0.65	0.57	0.2618	-0.50	1.80
**C5/C6**	TCA	85.15	1.33	85.31	1.72	-0.16	2.17	0.9414	-4.51	4.19
	GMA	20.20	0.36	19.60	0.47	0.60	0.59	0.3111	-0.58	1.79
**T9/T10**	TCA	47.05	0.73	46.24	0.97	0.81	1.22	0.5069	-1.63	3.26
	GMA	9.90	0.17	9.62	0.22	0.29	0.28	0.3045	-0.27	0.85
**T_{max}**	TCA	63.58	1.13	61.48	1.43	2.12	1.83	0.2536	-1.56	5.77
	GMA	24.73	0.58	22.83	0.75	1.91	0.95	**0.0497**	0.00	3.81

SE: Standard Error. CI: confidence interval. TCA: Total cross-sectional area. GMA: Gray matter area.

Supplementary Table 3: Total cross-sectional area (TCA) and gray matter areas (GMA) (in mm^2) at the intervertebral disc levels C2/C3, C3/C4, C4/C5, C5/C6, T9/T10, and T_{max} (level of the lumbar enlargement) in minors, middle-aged and elderly subjects. Means are least square means with adjustment for sex.

**Whole study population**		Group 1 Age:<18		Group 2 Age:18-65		Group 3 Age:>65		t-Test					
		Mean area (mm^2)	SE	Mean area (mm^2)	SE	Mean area (mm^2)	SE	Differ-ence G1-2	SE	p-value	Differ-ence G2-3	SE	p-Value
C2/C3	TCA	81.10	1.76	85.88	1.58	81.54	1.67	-4.78	2.34	**0.0457**	4.34	2.27	0.0615
	GMA	15.87	0.32	15.67	0.29	14.84	0.30	0.20	0.43	0.6382	0.83	0.41	**0.0495**
C3/C4	TCA	85.56	1.98	89.82	1.72	85.63	1.87	-4.26	2.60	0.1069	4.20	2.53	0.1021
	GMA	18.65	0.46	19.49	0.40	18.57	0.43	-0.84	0.60	0.1674	0.92	0.59	0.1219
C4/C5	TCA	87.76	2.17	89.91	1.89	86.80	2.05	-2.15	2.86	0.4539	3.11	2.77	0.2662
	GMA	20.26	0.53	20.23	0.46	19.94	0.50	0.03	0.69	0.9603	0.28	0.67	0.6738
C5/C6	TCA	85.60	2.05	86.63	1.68	83.25	1.83	-1.03	2.63	0.6969	3.38	2.46	0.175
	GMA	20.30	0.56	20.11	0.46	19.32	0.50	0.20	0.72	0.7873	0.79	0.68	0.251
T9/T10	TCA	47.34	1.24	47.94	0.88	44.64	0.95	-0.60	1.51	0.691	3.30	1.28	**0.0131**
	GMA	10.55	0.31	9.75	0.22	9.30	0.24	0.80	0.37	**0.0353**	0.44	0.32	0.166
T_{max}	TCA	62.63	1.78	63.52	1.44	61.38	1.49	-0.89	2.27	0.6957	2.15	2.06	0.3017
	GMA	25.81	0.91	23.52	0.76	22.53	0.79	2.29	1.18	0.0579	0.99	1.09	0.3654

SE: Standard Error. CI: confidence interval. TCA: Total cross-sectional area. GMA: Gray matter area.

Supplementary Table 4: Associations between anatomical variables and total cross-sectional cord areas (TCA) and gray matter areas (GMA) at the level of the lumbar enlargement (T_{max}) using Pearson correlation coefficients (whole study population).

**Whole study population**	**TCA_T_{max}**		**GMA_T_{max}**	
**Metric**	p-value	Pearson correlation coefficient	p-value	Pearson correlation coefficient
CAN_T_{max}_ap	0.643	-0.06	0.154	0.20
CAN_T_{max}_lat	0.774	0.04	0.477	0.10
CAN_T_{max}_area	0.982	-0.00	0.182	0.18
Product_CAN_T_{max}_ap*lat	0.966	0.01	0.254	0.16
VBW_T_{max}_lat	0.595	0.07	0.178	-0.19
VBH_T12	0.335	0.13	0.532	-0.09
McRae	0.918	0.01	0.683	0.06
ForMag_ap	0.712	-0.05	0.819	0.03
ForMag_lat	0.756	-0.04	0.848	0.03
ForMag_area	0.423	-0.11	0.957	0.01
TIV	0.063	0.25	0.040	0.28
Brain GM Volume	0.639	0.07	0.006	0.37
Brain WM Volume	0.012	0.34	0.157	1.93
Height	0.319	0.14	0.394	-0.12
Weight	0.060	0.26	0.158	-0.19
BMI	0.074	0.24	0.122	-0.21

CAN_T_{max}_ap: anterior posterior diameter of the spinal canal at the level T_{max}. CAN_T_{max}_lat: lateral diameter of the spinal canal at the level T_{max}. CAN_T_{max}_area: area of the spinal canal at the level T_{max}. . Prod_CAN_T_{max}_ap*lat: product of the anterior-posterior and lateral diameter of the spinal canal at the level T_{max}. VBW_T_{max}_lat: maximum vertebra body width at the level T_{max}. VBH_T12: middle vertebral body height of T12. McRae: McRae line (distance between Basion–Opisthion). ForMag_ap: anterior-posterior diameter of the foramen magnum. ForMag_lat: lateral diameter of the foramen magnum, ForMag_area: area of the foramen magnum. TIV: Total intracranial volume. GM: Gray Matter. WM: White Matter. BMI: Body Mass index.

p-values surviving the Bonferroni correction ($p<0.003$) are in bold face.

Supplementary Table 5: Associations between anatomical variables and total cross-sectional cord areas (TCA) and gray matter areas (GMA) at the level C4/C5 using Pearson correlation coefficients (only minors, n=18).

**Minors (n=18)**	**TCA_C4/C5**		**GMA_C4/C5**	
**Metric**	p-value	Pearson correlation coefficient	p-value	Pearson correlation coefficient
CAN_C4/C5_ap	0.110	0.39	0.042	0.48
CAN_C4/C5_lat	0.047	0.47	0.360	0.23
CAN_C4/C5_area	0.007	0.61	0.027	0.52
Prod_CAN_C4/C5_ap*lat	0.018	0.55	0.030	0.51
VBW_C4/C5_lat	0.173	0.34	0.843	0.05
VBH_C4	0.387	0.22	0.746	0.08
McRae	0.577	0.14	0.695	0.02
ForMag_ap	0.667	-0.11	0.748	-0.08
ForMag_lat	0.537	-0.16	0.880	-0.04
ForMag_area	0.442	0.19	0.306	0.26
Prod_ForMag_ap*lat	0.560	-0.15	0.760	-0.08
TIV	0.085	0.42	0.198	0.33
Brain GM Volume	0.357	0.23	0.377	0.22
Brain WM Volume	0.016	0.56	0.146	0.36
Height	0.099	0.40	0.369	0.23
Weight	0.029	0.51	0.175	0.33
BMI	0.033	0.50	0.138	0.36
Age	0.587	0.14	0.426	0.20

CAN_C4/C5 ap: anterior-posterior diameter of the spinal canal at the level C4/C5. CAN_C4/C5_lat: lateral diameter of the spinal canal at the level C4/C5. CAN_C4/C5_area: area of the spinal canal at the level C4/C5. Prod_C4/C5_CAN_ap*lat: product of the anterior-posterior and lateral diameter of the spinal canal at the level C4/C5. VBW_C4/C5_lat: maximum vertebra body width at the level C4/C5. VBH_C4: middle vertebral body height of C4. McRae: McRae line (distance between Basion–Opisthion). ForMag_ap: anterior-posterior diameter of the foramen magnum. ForMag_lat: lateral diameter of the foramen magnum. ForMag_area: area of the foramen magnum. Prod_ForMag_ap*lat: product of the anterior-posterior and lateral diameter of the foramen magnum. TIV: Total intracranial volume. BMI: Body Mass index. GM: Gray Matter. WM: White Matter.

p-values surviving the Bonferroni correction (p<0.0028) are in bold face.
